# Dental Workforce Trends and Emerging Workforce Challenges: Perspectives from a 15-Year Longitudinal Analysis of the Israeli Experience (2008–2023)

**DOI:** 10.3390/healthcare14131902

**Published:** 2026-06-30

**Authors:** Hagit Domb Herman, Dara Schwartz, Lena Natapov

**Affiliations:** Division of Dental Health, Ministry of Health, Jerusalem 9101002, Israel

**Keywords:** dental workforce, workforce planning, health workforce, workforce participation dentists, internationally trained dentists, dental specialists, hygienists

## Abstract

**Background/Objectives:** An effective dental workforce is essential to ensure timely, high-quality oral healthcare across health systems worldwide. Many countries are currently facing challenges related to workforce supply, professional mobility, and the alignment between training capacity and population needs. Using Israel as an empirical case, this study examines long-term trends in the national dental workforce and explores their relevance for international workforce planning. **Methods**: A retrospective longitudinal analysis was conducted using Israel’s National Health Professions Database (“Oskim”), supplemented by data on dental education and specialty training. Descriptive longitudinal analyses were performed to evaluate workforce trends over time, and inferential statistical analyses were used to assess differences in demographic and geographic workforce distributions. Workforce indicators were analyzed using internationally standardized metrics to enable comparison with OECD healthcare systems. **Results:** The number of licensed dentists increased substantially, while the number of actively practicing dentists grew more moderately, reflecting a widening gap between licensure and workforce participation. Between 2008 and 2023, the number of licensed dentists increased by 49.9%, compared with a 40.4% increase in the actively practicing workforce. Accordingly, the practicing dentist-to-population ratio increased only slightly, from 0.80 to 0.84 per 1000 population. Israel reported 0.84 practicing dentists per 1000 population in 2023, exceeding the OECD average of 0.70. However, despite exceeding the OECD average, the relatively modest increase in the practicing workforce suggests that higher dentist-to-population ratios may overestimate actual workforce capacity when workforce participation patterns are not taken into account. Internationally trained dentists accounted for approximately 75% of new licensees, highlighting the role of professional mobility in shaping workforce supply. The proportion of dental specialists remained relatively stable at approximately 10%. In addition, the number of licensed dental hygienists increased by 96.1% (from 1468 to 2879), while the number of newly licensed hygienists declined by approximately 43% (from 174 to 100 annually). **Conclusions:** Despite substantial growth in the number of licensed dentists, the practicing workforce and specialist capacity expanded only modestly, and geographic disparities persisted. The difference between growth in licensed dentists and the actively practicing workforce highlights the importance of incorporating participation measures into national monitoring and oral health planning policies. The Israeli experience illustrates how workforce expansion driven by internationally trained dentists may coexist with structural challenges in participation, specialization, and distribution. These findings highlight broader considerations for oral health workforce planning and may provide insights for other health systems facing similar demographic and staffing dynamics. Because indicators of oral health need, service utilization, disease burden, and workforce productivity were unavailable, the study could not determine whether the observed workforce supply was adequate to meet population oral health needs.

## 1. Introduction

The healthcare workforce is a vital component of any effective health system, and adequate human resources are essential for delivering accessible, high-quality health services. The dental workforce plays a central role in prevention, diagnosis, and treatment throughout the lifespan. As populations grow and age, many countries are facing increasing challenges related to workforce supply, professional mobility, training capacity, participation rates, and geographic distribution of providers. Increased attention has also been directed toward potential mismatches between population oral health needs and available workforce capacity.

Most recently, the WHO Global Oral Health Action Plan 2023–2030 called on countries to strengthen monitoring and evaluation of oral health workforce trends to support evidence-based workforce planning and ensure alignment between service capacity and population needs [1,2]. Regular assessment of dental workforce dynamics is therefore critical to ensure that workforce capacity keeps pace with evolving oral health demands [3]. Contemporary workforce planning models increasingly emphasize needs-based approaches that consider not only workforce supply, but also disease burden, service utilization, demographic trends, and population access to care.

Despite growing international attention to oral health workforce planning, comprehensive longitudinal analyses integrating workforce participation, specialization, international mobility, geographic distribution, and allied oral health professions remain limited in many health systems. Previous studies have often focused on isolated indicators such as dentist-to-population ratios or licensure counts without fully examining workforce participation, specialist capacity, or actual service availability [4,5,6]. In addition, many analyses rely on registration-based estimates that do not distinguish between licensed and actively practicing professionals, potentially limiting assessment of effective workforce capacity and access to care. As a result, important dimensions of workforce availability and long-term workforce sustainability may remain insufficiently evaluated for health policy planning [7].

Israel provides a relevant setting for examining these issues, given its evolving oral health system. Beginning in 2010, publicly funded dental services were gradually expanded for children, followed by expansion to adults aged 72 years and older beginning in 2019 [8,9]. The transition from a predominantly private dental care system toward broader public coverage has been considered an important innovation in oral health policy and service delivery. Israel’s combination of rapid demographic growth, substantial immigration, reliance on internationally trained dentists, and evolving licensure pathways provides a useful case study for examining how oral health systems adapt workforce planning strategies under changing conditions.

Parallel to these structural reforms, developments in Israel’s healthcare system have also contributed to increasing integration of advanced technologies and specialized approaches within oral healthcare. This is reflected in growing research and clinical innovation in areas such as predictive oral healthcare models, AI-driven diagnostics, temporomandibular disorders (TMD), bruxism assessment, and preventive oral healthcare models [10,11,12]. These developments may increase demand for appropriately trained dental professionals and specialists with competencies suited to evolving technologies, multidisciplinary care models, and population-based approaches to oral healthcare delivery.

Until the early 1970s, dental education in Israel was limited to a single national institution, the School of Dental Medicine at Hebrew University [13]. The establishment of the School of Dental Medicine at Tel Aviv University in 1972 expanded domestic training capacity; however, demand soon outpaced local capacity, prompting an increasing number of Israeli students to pursue dental education abroad. This trend intensified in the early 1990s, with the arrival of approximately 800 internationally trained dentists from the former Soviet Union, compared with a prior annual average of 150–200 new entrants [14]. As a result, the dental workforce expanded by 25.9%, far exceeding the 4% growth of the general population during the same period [14].

Before the early 1990s, both locally trained and internationally educated dentists with recognized degrees were permitted to practice in Israel without a national licensing examination. An amendment to the Dentists Ordinance in 1992 introduced mandatory national board examinations designed to evaluate competency before licensure [15]. A later amendment in 2016 created an alternative pathway allowing experienced practitioners to apply for exemption from the examination based on recent verified clinical practice [16]. While the number of licensed dentists has since increased, making Israel one of the countries with the highest dentist-to-population ratios in the OECD, the percentage of dental specialists has grown only modestly, rising from approximately 8% to 10%, despite increasing demand for specialized dental services [17].

Specialist training in Israel is governed by the Dental Practitioners Regulations of 1977, which established structured residency programs across nine recognized specialty fields [18]. These programs typically require between 3.5 and 5 years of training and are conducted in both hospital and academic settings. Board certification requires successful completion of both theoretical (Part I) and practical (Part II) examinations. Despite this well-established training framework, the number of specialists has not increased proportionally to population growth. Previous analyses have identified shortages in several specialties, including pediatric dentistry, oral medicine, and prosthodontics [19]. Demographic changes, including population growth and population aging, have further increased demand for specialized dental services, while specialist training capacity has remained relatively stable. Population aging, increasing chronic disease burden, and expanded public dental coverage may further increase demand for complex restorative, prosthodontic, oral medicine, and geriatric dental services in the coming years.

Dental hygienists constitute an additional and important component of the oral health workforce. In Israel, the profession has been formally regulated under the Dental Hygienists Regulations of 1977, which define licensure requirements and the scope of practice [20]. Licensure requires completion of an accredited educational program and successful completion of a two-part examination administered by the Ministry of Health. Dental hygienists play a central role in preventive and promotive oral health services, particularly within the School Dental Service and maternal and child health programs. Despite their importance in preventive care and community oral health, long-term trends in this professional group have received relatively limited attention.

Globally, increased attention has been directed toward how countries adapt oral health workforce strategies in response to demographic shifts, international mobility of health professionals, and evolving models of care delivery. International frameworks such as the WHO Global Strategy on Human Resources for Health: Workforce 2030 and the FDI World Dental Federation’s Vision 2030 emphasize coordinated approaches integrating education, regulation, and workforce planning to strengthen oral health systems [21,22]. Examining Israel’s experience within this broader international context enables assessment of how national approaches to training, licensure, and workforce organization align with global trends in oral health workforce development.

This study, therefore, aimed to examine long-term trends in Israel’s dental workforce between 2008 and 2023, including dentists, specialists, and dental hygienists, and to contextualize the findings within international workforce indicators and OECD benchmarks. Specifically, the study examined whether increases in the licensed dental workforce were accompanied by proportional changes in workforce participation, specialist capacity, and geographic distribution over time. By situating these findings within an internationally comparable framework, the Israeli experience may provide insights relevant to other health systems facing similar workforce planning challenges.

## 2. Materials and Methods

This retrospective longitudinal descriptive study examined national trends in the composition of Israel’s dental workforce from 2008 to 2023. The primary data source was the “Oskim” [23] computerized database of the Ministry of Health (MOH), which contains administrative records on licensed dental professionals. Supplementary data were obtained from the MOH periodic “Workforce in Healthcare Professions” reports [17], institutional records from Israel’s two accredited dental schools, and the MOH registry of dental specialty residency programs. The use of multiple national administrative and institutional data sources enabled evaluation of workforce trends across dentists, specialists, and dental hygienists over time.

Key workforce indicators were examined to evaluate changes in workforce composition and dynamics over time, including entry into the profession, workforce participation, and the demographic, geographic, and educational characteristics of dental professionals. To enable international comparison, selected indicators were contextualized using OECD benchmarks. The differences in workforce definitions, reporting methodologies, and data-collection standards across countries were taken into account when interpreting international comparisons.

Data regarding newly licensed dentists, dental specialists, dental hygienists, licensed dentists, and practicing dentists were obtained from national workforce reports and OECD databases. The source reports did not provide formal operational definitions for newly licensed dentists, dental specialists, dental hygienists, or licensed dentists; therefore, the terminology used in this study reflects the classifications reported in the original data sources. Practicing dentists were defined according to OECD-aligned workforce reporting standards as licensed dentists who reported performing clinical work for pay during the reference period. Nevertheless, these estimates may not fully reflect clinical workload, employment intensity, or actual service capacity.

Descriptive longitudinal analyses were conducted to identify trends and patterns throughout the study period. Inferential statistical analyses were also performed to assess the significance of observed differences in demographic and geographic distributions over time. The analytical approach was intended to complement descriptive workforce trend analyses by assessing whether selected observed differences were statistically significant.

Data management and preliminary analyses were conducted using Microsoft Excel (Microsoft Corp., Redmond, WA, USA). Statistical analyses were performed using IBM SPSS Statistics version 31.0.0.0 (IBM Corp., Armonk, NY, USA). Data extraction, cleaning, and analysis procedures were conducted using standardized predefined variables and coding definitions derived from the administrative datasets. To improve reproducibility and data consistency, extracted datasets and analytical outputs were cross-checked against published Ministry of Health workforce reports and institutional records. All analyses were conducted on anonymized administrative data, with no personal identifiers recorded or reported.

Chi-square tests were used to assess differences in categorical variables, including age distributions between dentists and dental hygienists and gender distributions between dentists and dental specialists. The Kruskal–Wallis test, a non-parametric method appropriate for skewed distributions, was used to evaluate differences in geographic workforce distribution over time, as regional workforce density measures were not normally distributed.

This study focused on administrative workforce data and regulatory analysis and did not involve human participants, clinical interventions, or identifiable patient information. The Ministry of Health Ethics Committee granted a formal exemption from IRB approval (MOH 039-2026) on 20 April 2026. Access to administrative workforce datasets was conducted in accordance with Ministry of Health data governance procedures, and all analyses were performed using anonymized aggregated workforce data.

## 3. Results

### 3.1. Licensing and Participation Trends

Between 2008 and 2023, the annual number of newly licensed dentists in Israel more than doubled, growing from 212 to 575. This surge contributed to a 49.9% increase in the total number of licensed dentists, which rose from 9130 to 13,679, and shifted the licensure-based dentist-to-population ratio from 1.31 to 1.39 per 1000 residents (+6.1%). This expansion occurred alongside a 34% growth in the general population, which expanded from 7.3 million to over 9.8 million individuals due to demographic shifts and immigration.

**Figure 1:**
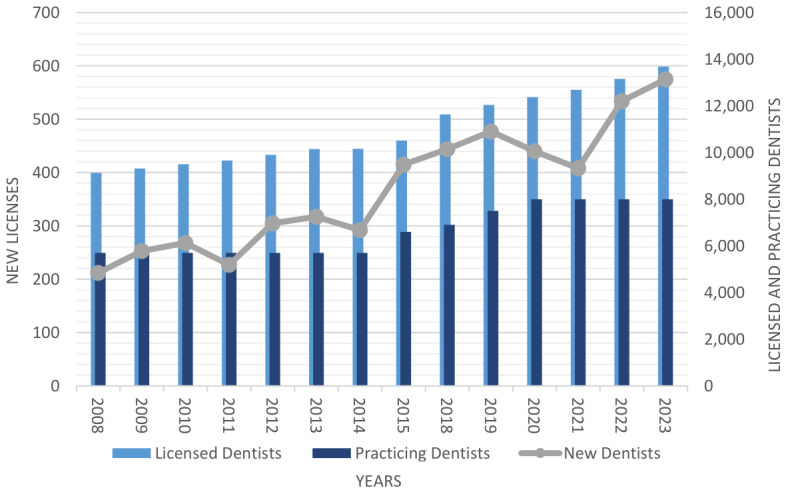
Licensed, practicing, and annual new dental licenses, 2008–2023.

In contrast, the actively practicing dental workforce grew more modestly, rising by 40.4% from approximately 5700 to 8000 practitioners. Consequently, the practicing dentist-to-population ratio increased slightly, from 0.80 to 0.84 per 1000 residents (+5.0%). This divergence demonstrates a clear gap between total licensure growth and active workforce participation (Figure 1).

**Figure 2:**
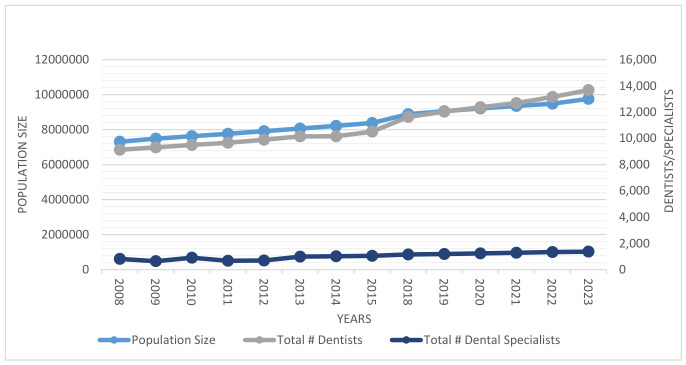
Dentists, specialists, and population size, 2008–2023.

While the general licensed workforce expanded faster than population growth, the specialist workforce remained limited. The specialist-to-population ratio changed minimally, rising from 0.11 to 0.14 per 1000 residents, and specialists consistently accounted for a stable 10% share of the overall dental workforce throughout the 15-year study period (Figure 2).

#### Workforce vs. Population

Growth in the licensed dental workforce generally exceeded population growth throughout the study period. However, specialist workforce growth remained comparatively modest. The specialist-to-population ratio increased only slightly, from 0.11 to 0.14 per 1000 population, while specialists consistently represented approximately 10% of the total dental workforce throughout the study period (Figure 2). This relative stagnation persisted despite overall workforce expansion and population growth.

### 3.2. International Benchmarking of the Dental Workforce

#### 3.2.1. Dentist Workforce Density

**Figure 3:**
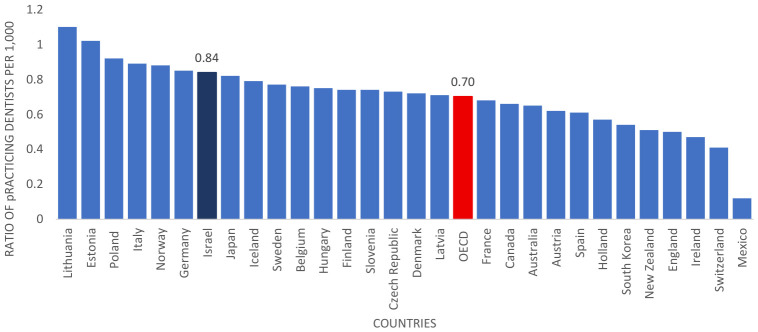
Practicing Dentists per 1000 population in Selected OECD Countries 2023 (or latest available year). Data sources: [15,22].

**Figure 4:**
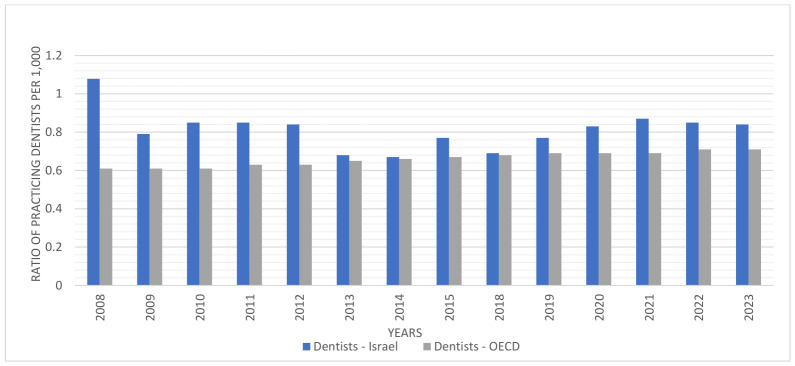
Practicing Dentists per 1000 population: Israel vs. OECD, 2008–2023. Data sources: [15,22].

Variations in practicing dentist density were observed across OECD countries, ranging from 1.19 per 1000 population in Lithuania and 1.06 in Estonia to 0.50 in England. Most countries reported densities between 0.70–0.90 per 1000 population, including Germany (0.85), Norway (0.86), and Italy (0.82). In 2023, Israel reported 0.84 practicing dentists per 1000 population, exceeding the OECD average of 0.70 and positioning Israel within the upper range of reported OECD workforce densities. Israel also consistently remained above the OECD average despite temporary declines in 2013–2014. The OECD average increased gradually from 0.61 to 0.71 during the same period (Figure 3 and Figure 4).

#### 3.2.2. Domestic Dental Training Output

In 2023, Israel’s domestic dental school graduate density was 1.78 per 100,000 population, substantially below the OECD average of 3.36 per 100,000 population. Israel therefore produced approximately half the OECD average number of domestic dental graduates relative to population size.

**Figure 5:**
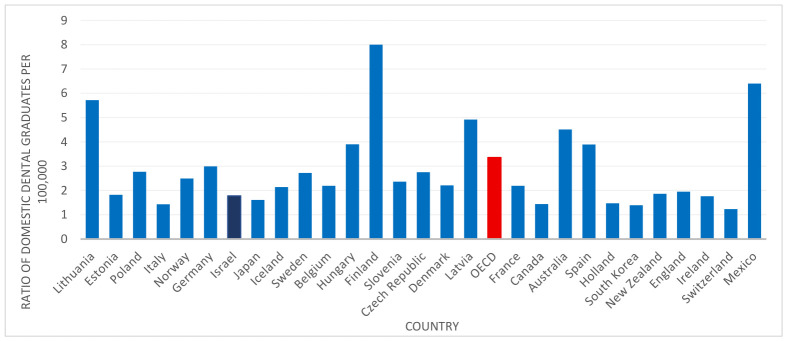
Domestic dental graduates in selected OECD countries, 2023. Data source: [15,22].

This pattern persisted when compared with countries of similar demographic scale. Sweden reported 2.72 graduates per 100,000 population and Hungary 3.9 per 100,000, both substantially higher than Israel’s rate. Among similarly sized countries included in the comparison, only Switzerland reported a lower graduate density (Figure 5).

### 3.3. Educational Trends

**Figure 6:**
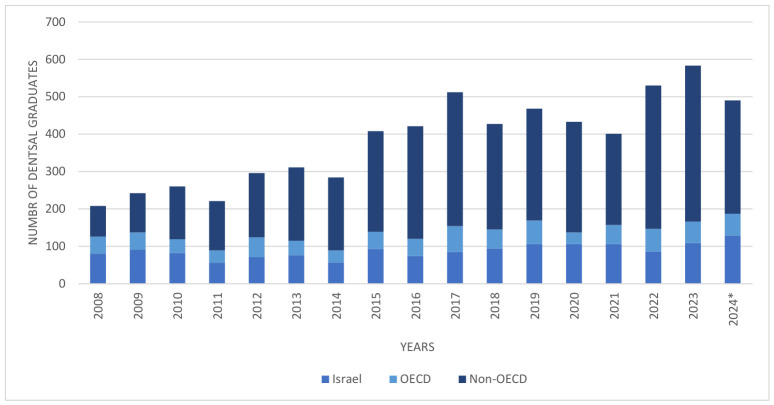
Trends in License Recipients by Educational Background (2008–2024). * 2024 data are preliminary.

Israel remained structurally reliant on internationally trained dentists throughout the study period. Graduates from non-OECD countries consistently accounted for the majority of new license holders, generally exceeding two-thirds of annual entrants into the workforce. The number of graduates from Israeli dental schools remained relatively stable at approximately 100–120 annually, with a projected increase to 129 in 2024. In 2023, Israeli-trained dentists accounted for 19% of new licenses, OECD-trained dentists for 16%, and non-OECD graduates for 65% (Figure 6).

### 3.4. Demographics and Geographic Distribution of Dentists

#### 3.4.1. Age and Gender Distribution

Dentists aged 45–64 constituted the largest workforce group throughout most of the study period, although their proportion gradually declined from 47% in 2010 to 39% in 2023. In contrast, the proportion of dentists younger than 44 years increased from 37% in 2018 to 41% in 2023, suggesting gradual workforce renewal in more recent years. Dentists aged 65 years and older remained relatively stable at approximately 18–20% of the workforce.

**Figure 7:**
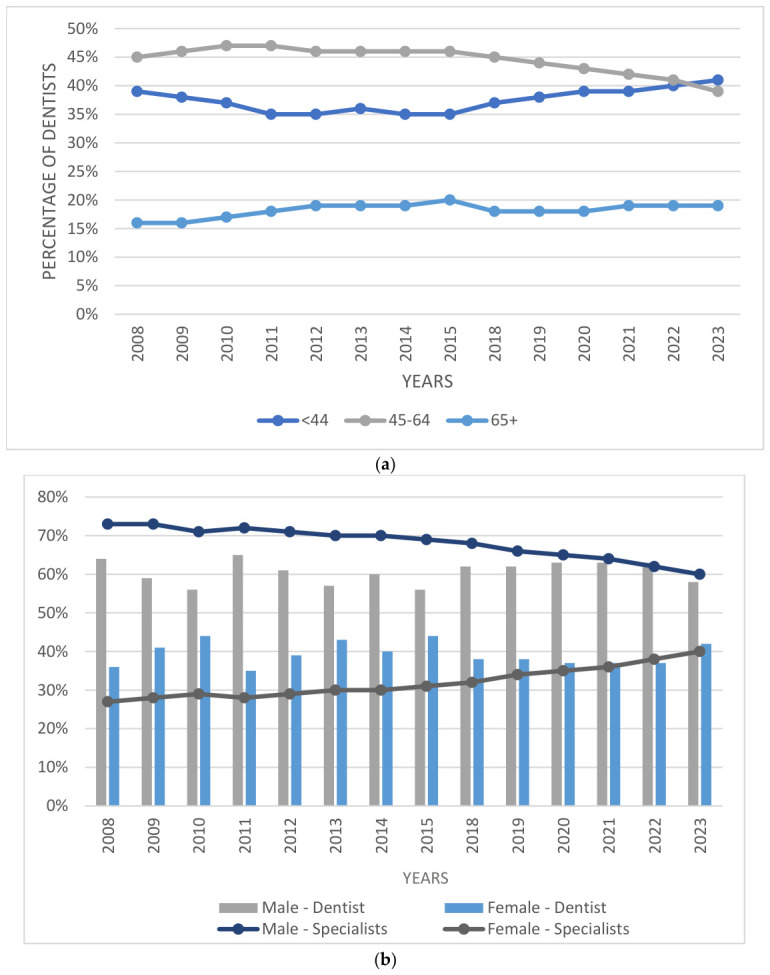
(**a**) Distribution of dentists by age group, 2008–2023, *p* < 0.001. (**b**) Gender distribution of dentists and specialists, 2008–2023, *p* < 0.001.

Female representation increased steadily over time. The proportion of female general dentists rose from 36% in 2008 to 42% in 2023, while among specialists it increased from 28% to 40%. Correspondingly, the proportion of male specialists declined from 73% to 60%. Chi-square analyses demonstrated statistically significant associations between year and both age distribution and gender distribution (*p* < 0.001) (Figure 7a,b).

#### 3.4.2. Geographic Distribution

Substantial geographic disparities in dentist-to-population ratios persisted throughout the study period. Tel Aviv consistently demonstrated the highest workforce density, reaching approximately 1.3 dentists per 1000 population, whereas the South consistently demonstrated the lowest ratios at approximately 0.4–0.5 per 1000 population. Jerusalem showed marked increases beginning in 2020, while the North demonstrated gradual improvement over time. Haifa and Central Israel remained relatively stable throughout the study period.

A Kruskal–Wallis test identified significant regional differences in workforce distribution (*p* < 0.001). Pairwise comparisons confirmed significantly higher dentist-to-population ratios in Tel Aviv compared with the South, Jerusalem, and the North. The South also demonstrated significantly lower workforce density than both Jerusalem and the North.

**Figure 8:**
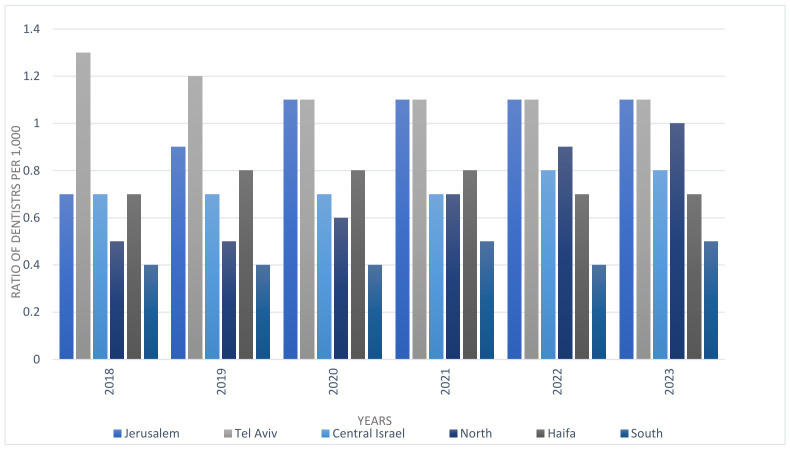
Geographic distribution of dentists, 2018–2023.

Furthermore, linear trend analyses between 2018 and 2023 demonstrated significant increases in workforce density in Jerusalem (*p* = 0.041), Central Israel (*p* = 0.042), and the North (*p* = 0.001), with the strongest increase observed in the North. In contrast, Tel Aviv demonstrated a modest but statistically significant decline over time (*p* = 0.041), while no significant changes were identified in Haifa or the South (Figure 8).

### 3.5. Allied Health Professionals: Dental Hygienists

#### 3.5.1. Licensing Trends

**Figure 9:**
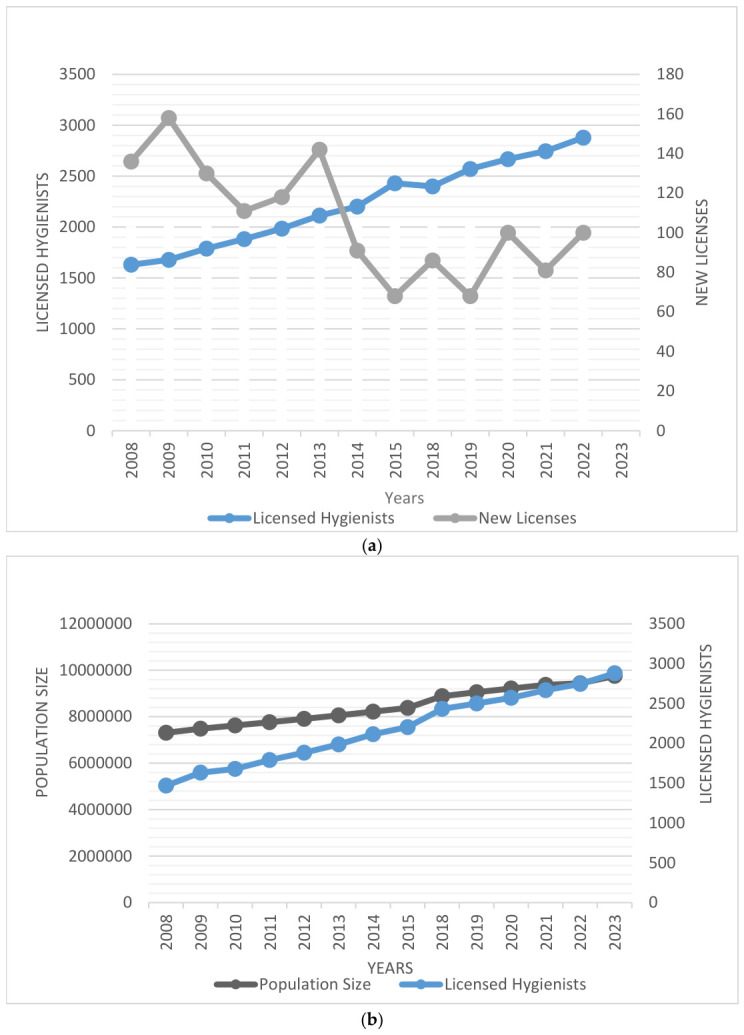
(**a**) Total licensed dental hygienists and annual new licenses, 2008–2023. (**b**) Number of licensed hygienists and population growth, 2008–2023.

The number of licensed dental hygienists in Israel nearly doubled, increasing from 1496 in 2008 to 2879 in 2023. This growth occurred alongside population expansion from approximately 7.3 million to over 9.5 million during the same period. Consequently, the ratio of licensed dental hygienists per 1000 population increased from 0.20 in 2008 to 0.28 in 2023. Despite overall workforce growth, the number of newly licensed dental hygienists per year declined from 174 in 2008 to 100 in 2023 (Figure 9a,b).

#### 3.5.2. Age Distribution

The dental hygiene workforce in Israel remained predominantly female throughout the study period, with women accounting for approximately 90% of licensed hygienists. Hygienists aged 31–44 consistently represented the largest workforce group, while those younger than 30 showed some decline over the time period. Mid-career hygienists aged 45–54 demonstrated steady growth, whereas hygienists aged 55 years and older continued to comprise a relatively small proportion of the workforce.

**Figure 10:**
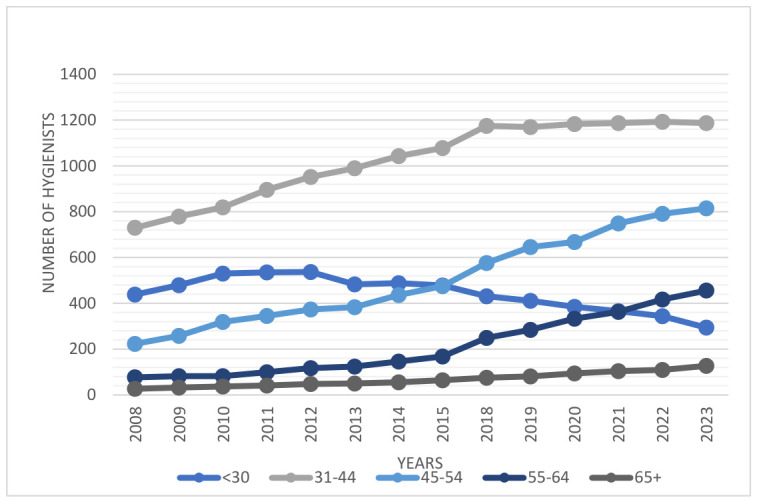
Distribution of hygienists by age group, 2008–2023, *p* < 0.001.

A chi-square test of independence demonstrated a statistically significant association between age group and year among dental hygienists in Israel (*p* < 0.001) (Figure 10).

## 4. Discussion

This study identified several long-term workforce trends in Israel, including divergence between licensed and practicing dentists, continued reliance on internationally trained practitioners, limited growth in specialist capacity, and persistent geographic disparities. These findings reflect broader structural workforce challenges facing oral health systems worldwide. Furthermore, they demonstrate the importance of workforce planning strategies that consider workforce participation, training infrastructure, professional composition, and geographic distribution.

### 4.1. Licensed and Actively Practicing Dentists in Israel

Between 2008 and 2023, the number of licensed dentists in Israel increased substantially, whereas growth in the actively practicing workforce was more moderate. Workforce expansion was driven primarily by internationally trained dentists obtaining licensure in Israel, while the number of graduates from Israeli dental schools remained relatively stable throughout the study period. At the same time, workforce capacity relative to population size increased more modestly, likely reflecting Israel’s rapid demographic growth.

Additional factors not examined in this study, such as retirement, part-time work, migration, workforce exits, and professional burnout, may also contribute to the gap between licensure growth and active workforce participation. Recent evidence from Israel has highlighted the potential impact of burnout, occupational stress, and work–life balance challenges among dental professionals, suggesting that workforce participation may be affected by factors beyond workforce supply alone [24,25]. However, information about these factors was not available in the administrative workforce datasets used in this study, and their role in the observed trends could not be assessed directly.

The divergence between licensed and actively practicing dentists demonstrates the importance of distinguishing between registration-based workforce counts and the effective dental workforce, as licensure data may include professionals who are retired, working abroad, employed in non-dental roles, or practicing part-time. Similar patterns have been reported in several OECD countries [26], emphasizing that licensure data alone may overestimate actual service capacity and should be interpreted cautiously in workforce planning and policy development. Incorporating workforce participation indicators into national workforce monitoring systems may improve estimation of effective workforce capacity and support more evidence-informed workforce planning.

Interpretation of international comparisons should be undertaken cautiously because workforce indicators are not uniformly defined across countries and reporting systems vary substantially. Accordingly, the international comparisons presented in this study are intended primarily as contextual benchmarks rather than direct measures of workforce adequacy or system performance. In particular, the definition of “practicing” dentists differs across jurisdictions, which may affect the comparability of workforce estimates. Consequently, the observed variation in workforce density across countries, ranging from 0.1 to 1.1 practicing dentists per 1000 population [17,26], should be interpreted with caution.

Although Israel appears to be within the mid-to-upper range of the countries examined, these comparisons may be influenced by differences in workforce definitions, data collection methods, and reporting practices. This variability highlights the limitations of relying solely on licensure-based workforce indicators when comparing oral health systems internationally. More standardized workforce measures that incorporate workforce participation, workload, and service capacity may provide a more meaningful basis for assessing workforce availability and informing workforce planning across countries.

### 4.2. International vs. Domestic Training

Domestic training patterns further illustrate the structural characteristics of Israel’s dental workforce. The number of domestically trained dental graduates per population remains relatively low compared with OECD benchmarks, reflecting limited national training capacity. This constraint contributes to sustained reliance on internationally trained dentists, many of whom are Israeli citizens who completed their education abroad, many in non-OECD countries. In 2023, approximately three-quarters of newly licensed dentists were graduates of institutions outside Israel, a proportion considerably higher than that reported in several other high-income countries, including the United Kingdom and Australia, where internationally trained dentists represent approximately 28% and 24% of new entrants, respectively [27,28].

While this model supports workforce supply and may help address short-term workforce shortages, long-term reliance on internationally trained professionals raises important considerations regarding workforce sustainability and domestic training capacity. These findings highlight the importance of balancing the expansion of domestic educational capacity with effective licensure and integration pathways for internationally trained practitioners. The Israeli experience may provide insights for other health systems that increasingly rely on internationally educated oral health professionals to supplement the domestic workforce supply.

### 4.3. Dental Specialists

Despite the overall increase in the number of dentists, the proportion of dental specialists remained relatively stable at approximately 10% of the workforce throughout the study period. This finding is notable given the expansion of publicly funded dental services for children and older adults in Israel [8,9], populations that often require more complex or specialized care. Similar concerns regarding the balance between general dentists and specialists have been reported in other countries, emphasizing that workforce planning must consider not only overall workforce size but also professional composition and skill mix [5,29,30]. These observations highlight the importance of periodically reassessing specialty workforce needs and aligning specialist training capacity with projected service demands. Expanding residency positions in selected specialties may help strengthen access to advanced oral healthcare services.

### 4.4. Geographic Disparities

Geographic disparities further illustrate structural challenges in workforce distribution. Dentist density in Israel remains higher in major metropolitan areas and lower in peripheral regions, consistent with international patterns. Comparable urban–rural disparities have been documented in several countries, including Australia, the United States, and the United Kingdom [31,32,33,34]. These findings suggest that market-driven distribution alone may be insufficient to ensure equitable access to oral health services and that targeted policy interventions may be required. Potential approaches to address these disparities include targeted incentives, expansion of training opportunities in peripheral regions, and workforce retention strategies.

Although the study identified important workforce imbalances, it did not directly assess whether workforce distribution and composition were adequately aligned with population oral health needs across demographic groups or geographic regions. Assessment of workforce adequacy in relation to disease burden, unmet treatment needs, service utilization, geographic accessibility, oral health outcomes, and age-related treatment demand was beyond the scope of the present study and the available administrative datasets. Consequently, the findings should be interpreted primarily as workforce supply indicators rather than direct measures of workforce adequacy or accessibility. Future research integrating workforce data with population oral health indicators may help better evaluate alignment between workforce capacity and population need.

Demographic trends within the profession also reflect broader global developments. The increasing proportion of women in dentistry in Israel mirrors international trends [35,36,37]. While the growing participation of women contributes to workforce diversity, variations in career trajectories, working patterns, and specialty preferences may influence overall workforce capacity and service delivery [38]. In addition, the coexistence of a large mid-career cohort alongside increasing numbers of early-career practitioners highlights the importance of long-term workforce sustainability and succession planning within the profession [39].

### 4.5. Dental Hygienists

Trends in the dental hygienist workforce may have important implications for preventive and community-based oral health services. Although the total number of licensed hygienists increased substantially during the study period, the number of newly licensed hygienists declined. This pattern may indicate a potential slowdown in workforce replenishment over time. International evidence suggests that strengthening education pathways and expanding the roles of allied oral health professionals can improve preventive service capacity and enhance the efficiency of oral health systems [40,41]. Given the increasing emphasis on prevention-oriented and team-based oral healthcare delivery models, strengthening the dental hygienist workforce and expanding educational capacity may help improve preventive service delivery and reduce disparities in access to care.

Policy measures to expand dental hygienists’ educational capacity may help address future workforce shortages and strengthen preventive oral healthcare services. In this context, Israel is currently considering developing a bachelor’s degree program in dental hygiene. This may enhance clinical competencies, strengthen professional recognition, and support greater integration into interdisciplinary healthcare teams.

The findings of this study align with the priorities outlined in the WHO Global Oral Health Action Plan (2023–2030), which emphasizes the development of sustainable, well-distributed, and competency-based oral health workforces supported by robust data systems [1]. The observed patterns in workforce participation, training capacity, specialist availability, and geographic distribution reflect challenges identified internationally. The Israeli experience provides insights into how structural workforce factors may influence long-term workforce composition and capacity.

The COVID-19 pandemic likely influenced oral healthcare systems and workforce dynamics during the latter years of the study period, including in Israel. Dental education and clinical training programs experienced substantial disruptions, including the temporary suspension of clinical activities and the transition to online and blended learning models. While online learning supported educational continuity and accessibility of theoretical instruction, concerns were raised regarding reduced hands-on clinical exposure and limitations in patient-based training [42]. Blended learning approaches accelerated integration of digital learning and digital health technologies into dental education and training. Continued evaluation of how such approaches can effectively support both theoretical and clinical training may help inform future workforce and educational planning.

This period also highlighted the importance of preparedness and continuity planning within oral healthcare systems, particularly in dental settings characterized by close patient contact and aerosol-generating procedures [43]. In Israel, integrating oral healthcare into the broader healthcare system facilitated national monitoring, the implementation of infection-control protocols, and the continuation of safe dental care [43]. Previous Israeli data demonstrated low SARS-CoV-2 transmission rates in dental settings when infection-control measures were maintained [44]. These experiences underscore the importance of incorporating emergency preparedness and infection-control training into future oral health workforce planning and professional education.

### 4.6. Study Limitations

This study has several limitations. The analysis relied primarily on administrative licensure data, which may not fully reflect effective workforce capacity. In addition, the study lacked direct indicators of service utilization, patient outcomes, and clinical workload distribution, limiting the ability to assess the relationship between workforce composition and actual service delivery capacity.

Furthermore, the analyses mainly relied on aggregated annual workforce indicators and demographic data from national administrative workforce reports. Information about retirement patterns, employment intensity, migration, workforce exits, career changes, and other factors affecting workforce participation was not available in the source data. As a result, although the study found a difference between licensed and actively practicing dentists, it was not possible to analyze the reasons behind these differences in workforce participation.

International comparisons should also be interpreted cautiously because workforce definitions and reporting systems are not fully standardized across countries. In particular, variations in the operational definition of “practicing dentists” may affect the comparability of workforce estimates and limit direct cross-country comparisons. These limitations should be considered when interpreting cross-country differences in workforce availability and workforce density indicators.

Nevertheless, by integrating multiple workforce indicators and situating the findings within an international context, this analysis provides insights into how workforce participation, training capacity, professional composition, and geographic distribution interact to influence oral healthcare service capacity. The findings are consistent with broader evidence supporting needs-based workforce planning models that incorporate workforce participation, demographic trends, regional distribution, professional skill mix, and projected service demand rather than relying solely on licensure-based workforce estimates.

## 5. Conclusions

Taken together, these findings demonstrate that growth in the number of licensed dental professionals does not necessarily translate into proportional increases in workforce participation, service capacity, or access to care. Workforce participation, training capacity, professional composition, and geographic distribution all contribute to effective workforce capacity and should be considered alongside licensure-based workforce estimates when evaluating oral health workforce systems.

The findings highlight the importance of workforce planning policies that extend beyond licensure-based workforce counts and incorporate indicators of active clinical participation, specialist availability, and regional workforce distribution. Policy measures such as strengthening national workforce monitoring systems, expanding specialist training positions, improving incentives for practice in underserved regions, and supporting integration of internationally trained dentists through structured licensure and workforce transition pathways may help improve workforce capacity and access to care. In addition, continued investment in preventive and community-based oral healthcare services may help support more equitable and sustainable oral healthcare delivery.

Israel’s experience may provide relevant insights for other health systems facing demographic change, international professional mobility, and transitions in oral healthcare delivery models, particularly in settings expanding publicly funded dental services. However, differences in healthcare organizations and workforce regulations across countries should be considered when assessing international applicability. At the same time, some evidence gaps remain regarding the relationship between workforce supply and population oral health needs. The absence of data on oral health needs, service utilization, disease burden, and workforce productivity prevented an assessment of whether the available dental workforce was sufficient to meet the population’s oral health needs. Future research should further examine workforce productivity, clinical workload, service utilization, and the relationship between workforce composition and oral health outcomes in order to better inform long-term workforce planning and policy development.

## Data Availability

The data presented in this study are available on reasonable request from the corresponding author. The data are not publicly available due to institutional and administrative restrictions associated with Ministry of Health databases.
